# Ready to Eat Food: A Reason for Enhancement in Multidrug Resistance in Humans

**DOI:** 10.34172/apb.2024.023

**Published:** 2024-01-13

**Authors:** Sheetal Negi, Sarika Sharma

**Affiliations:** ^1^Department of Microbiology, Lovely Professional University Phagwara (Punjab), India.; ^2^Department of Sponsored Research, Division of Research & Development, Lovely Professional University Phagwara (Punjab), India.

**Keywords:** Ready-to-eat (RTE), Drug resistance, Food, Pathogens, Multi-drug resistance

## Abstract

The increasing trend of consuming ready-to-eat (RTE) food has become a global phenomenon, and this has raised concerns about the potential negative impacts on human health. Recent studies have shown a correlation between the consumption of RTE foods and the expansion of multidrug resistance (MDR) in humans. MDR is a significant challenge in the effective theory of infectious diseases, as it limits the effectiveness of antibiotics and other drugs used in therapy. Consumption of RTE food contribute to the development of MDR in humans. Additionally, there are potential risks of consuming RTE food contaminated with antibiotic-resistant bacteria, which can cause severe health consequences. The article highlights the need for awareness campaigns on the potential hazard related to the ingestion of RTE food and the importance of responsible and safe food production practices. It also recommends the need for regulatory bodies to establish strict guidelines for the production and distribution of RTE food to ensure that they are free from harmful contaminants and that their consumption does not lead to the development of MDR in humans. Overall, this article provides a comprehensive analysis of the potential negative impacts of RTE food consumption on human health and emphasizes the need for a more cautious approach to food consumption to protect public health.

## Introduction

 Ready-to-eat (RTE) food has become increasingly popular in recent years and become an integral part of our busy and fast-paced lifestyles. These foods are readily available and require little to no preparation before consumption.^1^ Examples of RTE foods include sandwiches, salads, pre-cooked meals, and packaged snacks. These foods are often pre-cooked or prepared and require minimal preparation before consumption.^2^ While RTE foods may be convenient, they have also been associated with the beginning and spreading of multi-drug resistant (MDR) bacteria in humans. Consumption of RTE foods has been known as an important hazard cause for the transmission of MDR bacteria to humans.^3^

 MDR bacteria are bacteria that have developed resistance to multiple types of antibiotics. These bacteria pose a significant public health threat, as they are difficult to treat and can cause intense and sometimes serious infections. The emergence and spread of MDR bacteria are due to various factors, including the large-scale use of antibiotics in agriculture and food production, the global movement of people and food products, and the overuse and misuse of antibiotics in human medicine.^4^

 RTE foods can be a source of MDR bacteria in several ways. First, the processing and handling of RTE foods can lead to the contamination of these foods with MDR bacteria.^5^ Secondly, the utilization of antibacterial drug in agriculture and food production can contribute to the development of MDR bacteria, which can then contaminate RTE foods. Finally, the consumption of RTE foods that have been contaminated with MDR bacteria can lead to the transportation of these bacteria to humans.

**Figure 1:**
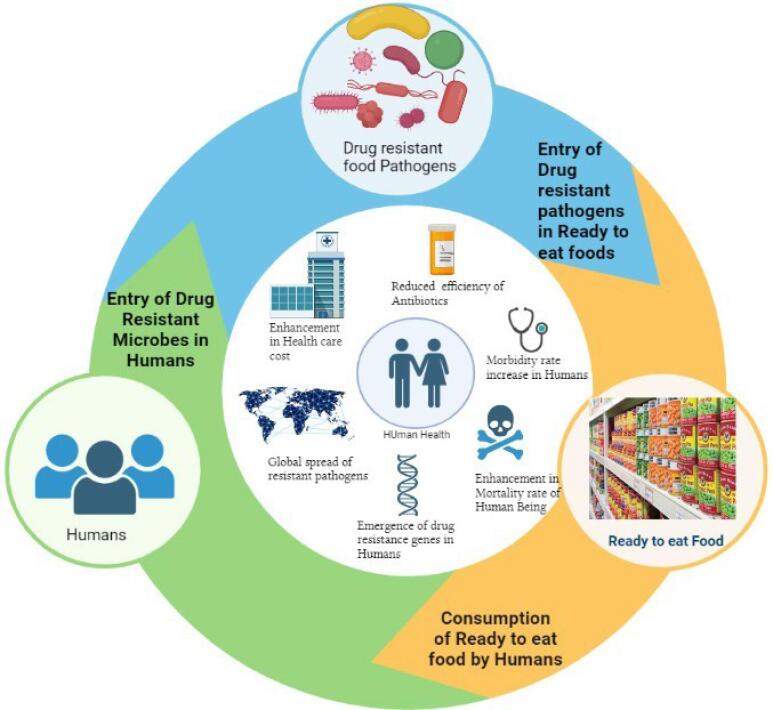
Entry of drug resistance food pathogens from food to human and its effects on human health

 The utilization of antibiotics in agriculture and food production has been linked to the beginning of MDR bacteria in food products (Figure 1), including RTE foods.^6^ The more use of antibiotics in animal husbandry and agriculture has led to the development of antibiotic-resistant bacteria, which can contaminate food products and cause infections in humans. Additionally, the processing and handling of RTE foods can also lead to the transmission of MDR bacteria.^7^

 Several types of MDR bacteria have been associated with RTE foods, including methicillin-resistant *Staphylococcus aureus* (MRSA), Salmonella, and *Escherichia coli*. MRSA is a type of bacteria that is resistant to many types of antibiotics and can lead to soft tissue infections, skin infections, pneumonia, and bloodstream infections.^4^ Salmonella is a type of bacteria that can cause food poisoning, with symptoms including diarrhea, fever, and sepsis in humans.^8^ *E. coli* is a type of bacteria that can cause severe gastrointestinal illness, with symptoms ranging from mild to severe diarrhea, abdominal pain, and fever.^9^

 The link between RTE foods and MDR bacteria is a significant public health concern. The emergence and dissemination of MDR bacteria are challenging to control, and the evolution of new antibiotics is slow and costly. Therefore, it is crucial to take steps to reduce the consumption of RTE foods and to implement measures to prevent the transmission of MDR bacteria in food products.^4^

## Ready to eat food

 RTE foods are convenient and popular due to their minimal preparation requirements and short cooking times. They cater to busy lifestyles and are especially favored during the COVID-19 pandemic.^10^ RTE foods include items like frozen snacks and bakery products.^11^ RTE foods, such as RTE cereal, are known for their nutritional adequacy and are particularly beneficial for children and adolescents. Studies show that consuming RTE cereal is associated with better nutrient intake.^12^ Shelf life is a key factor contributing to the popularity of RTE foods, especially in regions like Asia, Europe, and America. Brown rice, rich in dietary fibers and nutrients, has a shorter shelf life and longer cooking time compared to white rice. However, the development of RTE brown rice products overcomes these limitations.^13^ RTE fish is preserved in chilling and freezing conditions, maintaining its quality for extended periods.^14^ In metros and urban areas, RTE food is a trend, catering to individuals with hectic schedules who lack the time to prepare meals. RTE cereals save time and are a healthy breakfast option.^15^ RTE frozen fruits and vegetables retain their nutritional value and can be easily transported.^16^ Additionally, RTE foods are cost-effective. Despite their convenience, RTE foods have drawbacks. They often contain added ingredients like preservatives, artificial colors, sugar, and fats, leading to health issues such as obesity, type 2 diabetes, and heart disease.^17^ High trans-fat content in ultra-processed foods can increase the risk of cardiovascular diseases.^18^ RTE vegetables can absorb harmful chemicals, posing risks like bladder cancer and reproductive problems.^19^ Excessive fluoride in RTE infant foods can lead to dental fluorosis.^20^

 RTE raw fish, like sushi and sashimi, is consumed for its nutritional quality but carries biological and chemical hazards. Contaminants in fish tissue can impact human health.^21^ RTE salad dishes are susceptible to contamination from handling, dirty water, and cross-contamination between cooked and raw ingredients.^22^ Ingesting RTE salads and sprouts can lead to foodborne illnesses, including non-tuberculosis mycobacteria (NTM) infections in immunocompromised individuals. NTM can be present in soil, water, and fertilizers and are transmitted to fresh fruits and vegetables.^23^ RTE foods can also be a reservoir of antibiotic-resistant bacteria, which can transfer antibiotic-resistant genes to the bacteria in the human gut. This poses health risks as these antibiotic-resistant genes can affect human health.^24^

## Pathogens associated with RTE food

 RTE foods become most appropriate medium for the growth of pathogenic microorganisms which causes threat to public health. According to World Health Organization (WHO) there are more than 200 diseases spread by foods.^25^ Some major food pathogens are:

###  Listeria monocytogenes

 A Gram-positive pathogenic bacteria called Listeria monocytogenes causes listeriosis which is a food borne illness. This microbe is prevalent in RTE meat because of the poor hygienic conditions, exposure of the meat during sale and storage conditions of meat.^26^ Listeriosis mainly affect the people whose immunity is weak i.e., immunocompromised patient, old people, pregnant women and neonates.* Listeria* mostly affect the pregnant women. During the infection pregnant women suffer from mild febrile illness. Maternal listeriosis can passed onto the fetus where it causes neonatal listeriosis which has a rate of high mortality of 25%-30% depending upon the gestational age.^27^

###  H. pylori

*Helicobacter pylori *is a foodborne pathogen present in RTE food like hamburger, minced beef, RTE tuna meat etc. Various samples contained *H. pylori* like hamburger (1.42%) and in minced beef (12.5%).^28^ TheDNA of* H. pylori* was found in 36% raw chicken and 44% in RTE raw tuna meat samples.^29^ Furthermore 550 samples of RTE foods, out of 74% of samples detecting *H. pylori* are; restaurant salad (30%), olive salad (36%), soup (22%) and fruit salad (28%) these samples were contaminated with *H. pylori*.^30^ *H. pylori* was also detected in 60 out of 300 RTE food samples like hamburger (8.33%), meat sandwiches (10%), RTE fish (15%), vegetable sandwiches (18%), minced meat (32%) and chicken sandwiches (5%).^31^ There are more prevalence of* H. pylori* in RTE foods due to the contamination in hygiene and also during the handling of RTE food, preparing and packing of RTE food so, the *H. pylori* when transmitted to humans can cause disease in humans such as chronic disorders of upper gastrointestinal tract, gastric ulcer disease, gastric adenocarcinoma, low grade B cell MALtoma ( Mucosa associated lymphoid tissue lymphoma of the stomach) etc. it can also cause iron deficiency anemia, autoimmune thyroid disease, rosacea, thrombocytopenic purpura, idiopathic urticarial and extra-gastric disorders like coronary heart diseases. 80% of population in developing countries including young children are *H. pylori *positive whereas in industrialized countries the presence of *H. pylori *remains 40% and present in high numbers in adults and old people rather than young children and adolescents.^32^

###  Salmonella enterica

*Salmonella enterica *is a food borne pathogen and found in RTE meats. 300 samples of RTE meat was tested that includes beef, chicken, mutton, chevon, pork, guinea fowl. The street RTE meat are contaminated in many ways by poor handling of meat or not washing hands, do’ not drink, smoke or eat while selling the meat it increases the risk of contamination.^33^ *Salmonella enterica* strains are a common reason for concern in the field of food safety as they are the primary culprit in outbreaks of bacterial food poisoning around the world.^34^ Sushi and sashimi, two well-known RTE foods, have been linked to salmonellosis epidemics.^35^ The importance of RTE salad vegetables in the human diet is rising. Inadequate cleaning during processing, however, can result in some foodborne infections, such salmonellosis, because they are eaten raw. In the study on foodborne disease outbreaks, Salmonella spp. were the pathogens most commonly reported (41.0%) which was published by the Public Health Laboratory Service (PHLS) Communicable Disease Surveillance Centre (CDSC) during 1992-2000. 83 (5.5%) of the 1518 foodborne outbreaks of infectious intestinal illness were related to the eating of fruit or salad vegetables.^36^

###  Klebsiella pneumonia 

*Klebsiella pneumonia* is a food borne pathogen present in RTE meat [Luncheon-meat] and it causes diarrhea, septicemia in humans and the infection caused by *K. pneumonia *in humans is known as nosocomial infections.^37^ *Klebsiella* also responsible for pneumonia, lower biliary, urinary tract disease, blood stream infections, pyrogenic liver abscess, meningitis and intra-abdominal infections in humans.^38^ 350 meat samples were collected Mansoura city, Egypt from which 44 (12.6%) *K. pneumonia* species were isolated from RTE meat samples.^37^

###  Aeromonas spp

*Aeromonas spp*is found in RTE seafood products and seafood products are at the highest risk for foodborne outbreaks and it is because people are more interested towards RTE seafood in industrialized countries.* Aeromonas* bacteria is an aquatic bacterium and is a human pathogen due to presence in all types of food but especially in seafood as it causes spoilage. Some of the *Aeromonas spp* can grow unrepressed in food during the course of refrigeration under modified packaging atmosphere, NaCl and pH concentration. Most clinical strains come from a subset of four species that are more frequently linked to infections in humans. A*eromonas caviae, Aeromonas dhakensis, Aeromonas veronii biovar sobria, and Aeromonas hydrophila* causes acute gastroenteritis, potentially fatal infections like septicemia, and meningitis.^39^

###  Escherichia coli 

*Escherichia coli *belongs to a family of Enterobacteriaceaea foodborne pathogen in RTE meat and food that are sold in street causes food borne illnesses in humans by producing Shiga toxins and causing illness and deaths. Presence of *E. coli *in RTE meats include eight samples of chicken (16%), three samples of pork (6%), ten samples of chevon (20%), four samples of beef (8%), nine sample of guinea fowl (18%) and four samples of mutton (8%) were contaminated by *E. coli*.^40^ A study conducted in Egypt showed the presence of* E. coli *in total 90 samples of RTE sandwiches of meat and chicken in which 15 of each samples were taken from both meat and chicken, from meat shawarma (33.3%), kofta (40%) and hawawshi (46.7%) and from chicken shiesh tawook (26.7%), panee (33.3%, shawerma (33.3%).^41^ A study was conducted in Himachal Pradesh in which 265 RTE milk and milk-related products altogether were gathered to check the presence of *E. coli.* 65 samples of burfi collected from which four (6.15%) were positive for *E. coli*, 54 samples of paneer from which four (7.40%) were positive, 42 samples of cream roll and 12 of curd from which no *E. coli* was detected, 31 samples of milk tea out of which three were positive, 47 samples of ice cream out of which one was positive and 14 samples of pasteurized milk out of which one was positive for *E*. *coli* strain. Several strains of *E. coli* can lead to hemolytic uremic syndrome and diarrhea.^42^

###  Bacillus cereus 

*Bacillus cereus* has been screened from RTE foods. B. cereus can result in food poisoning at relatively low concentrations, if a food contains higher amount of *B. cereus* than 10^3^ than it is considered unsafe for consumption.^43^ 860 RTE food samples were taken from China which include rice/noodle, cooked meat and cold vegetables dishes in sauce. From all these samples 302 samples were positive for *Bacillus,* including 59 of 119 the rice/noodles samples (50%), 19 out of the 85 cold vegetable dishes in sauce samples (22%) and 224 out of the 656 cooked meat samples (34%) so, this indicate that RTE foods are harmful for consumption as it causes severe food safety problems.^44^

###  Staphylococcus aureus 

*Staphylococcus aureus *is found in RTE items such as sushi and sashimi which causes food poisoning and Salmonellosis outbreak to the people who consume it. These RTE food are prepared with bare hands so level of contamination with foodborne pathogens is high in these foods. In a study, 200 sample of sushi and 51 of sashimi was taken out of which Staphylococcus was detected in 26%.^35^

## Drug resistance in RTE food pathogens

 Drug resistance in RTE food pathogens is a growing concern in the field of food safety. RTE foods are those that do not require cooking before consumption, such as deli meats, salads, and fresh fruits. Pathogens such as *Salmonella, Listeria, and E. coli* can contaminate these foods during production, processing, and packaging. Utilizing antibiotics in agriculture and animal husbandry has been identified as a major contributor to the emergence of drug-resistant forms of these diseases. When these drug-resistant pathogens infect humans, they are challenging to treat with conventional antibiotics, raising the danger of serious illness and death. Drug resistance limit the use of drugs and hinder the treatment process for the patients. Drug resistance is mainly associated with the changes in the gene like gene amplification, site mutations and deletions. Resistance against any drug involve decreasing the effectiveness of the drug as the drug was administered frequently so the pathogen developed resistance against that drug and hence the effectiveness of the drug is decreased.^45^ The mechanism of drug resistance includes drug target modification, active drug efflux, drug uptake restriction, and drug inactivation.^46^ The antibiotic resistant pathogen can also transfer their resistance genes to other microbes and humans via food chain. RTE products are potential vehicle for spreading antibiotic-resistant pathogenic bacteria. There are more chances of survival and growth of microorganisms in RTE foods because of handling and processing of food in unhygienic conditions and no requirement of.^47^ It has been reported globally that antibiotic resistant bacteria cause death of 700 000 people and the mortality rate increases to 10 million by the year 2050.^48^ Antibiotic resistance in *Listeria monocytogenes* also found in RTE Foods in Turkey. Salmonella one of the food-borne diseases that carries a significant danger to human health and has a remarkable global reach are resistant to multiple drugs.^49^ *S. aureus *species are frequent human and animal infections that were first identified as penicillin-resistant in 1948. These resistant microorganisms are crucial for dairy products.^50^ Birds and mammals’ intestinal tracts frequently harbor enterococcispp., which are recognized as sign of enteric contamination in food. These pathogens can withstand unfavorable conditions like saline waters, low or high pH, and temperature, which shows that resistant enterococcican play a significant part in the expansion of diseases in the community. Additionally, resistantenterococcimay indirectly cause harm by passing on their resistance genes to strains that have been adapted to live in humans.^51^

Table 1 summarizes the list of major drug resistant food pathogens from various ready to eat food items.

**Table 1:** List of food pathogens resistant to drugs

**Name of the pathogen**	**Drug resistance**	**References**
*Salmonella enterica*	Nalidixic acid, neomycin, sulfonamides, tetracycline (TET)	^36^
*Listeria monocytogenes*	TET, trimethoprim-sulfamethoxazole, ampicillin (AMP), erythromycin, penicillin G (PEN), TET	^52,53^
S*taphylococcus aureus*	TET, cefoxitin, gentamicin, amikacin, vancomycin, erythromycin, ciprofloxacin, trimethoprim-sulfamethoxazole, chloramphenicol, cefoperazone, penicillin, erythromycin, clindamycin, azithromycin, trimethoprim/sulfamethoxazole	^54,55^
*Yersinia enterocolitica*	AMC, cephalothin, amoxycillin, TET, imipenem, gentamycin, piperacillin, amikacin, aztreonam, ciprofloxacin	^56^
*Escherichia coli*	AMC, ciprofloxacin, chloramphenicol, cotrimoxazole, imipenem, ertapenem, cefepime, cefoperazone sulbactam, gentamicin, amikacin, ciprofloxacin	^57,58^
*Bacillus cereus*	Cefixime (CFM), PEN, amoxicillin/clavulanic acid (AMC), ceftazidime (CAZ), AMP, ceftriaxone (CTR), cefotaxime, clindamycin	^59^
*Klebsiella pneumoniae*	AMP, cefepime, cefuroxime, cefotaxime, meropenem, ciprofloxacin, gentamicin	^58^
*Helicobacter pylori*	Amoxicillin, AMP, metronidazole, TET	^30^
*Aeromonas hydrophila*	Erythromycin, AMP, AMC, azithromycin, gentamicin, streptomycin, sulfisoxazole, TET trimethoprim/sulfamethoxazole	^60^

## Genes associated with drug resistance in RTE food pathogens

 Genes associated with drug resistance in RTE food pathogens are of great concern due to the potential for serious health consequences. These genes encode proteins that confer resistance to antibiotics and other drugs, making infections caused by these pathogens more difficult to treat. Commonly identified genes linked to drug resistance in RTE food pathogens include those for tetracycline, fluoroquinolones, and aminoglycosides. These genes can be present in the bacterial genome or acquired through horizontal gene transfer from other bacteria. The spread of drug-resistant RTE food microorganisms is a significant public health issue, emphasizing the importance of food safety measures and antibiotic stewardship in both human and animal populations. In one study, antibiotic resistance genes were found in bacterial strains from RTE foods, including chloramphenicol resistance genes in *S. maltophilia* and *Pseudomonas* spp., gentamycin resistance genes in *Acinetobacter* spp. and *Pseudomonas* spp., and tetracycline resistance genes in Enterobacteriaceae spp. Additionally, various other resistance genes like ESBLs, OXA, carbapenemases, and plasmid-mediated AmpC genes were detected.^61^

 In 244 RTE foods, 267 *S. aureus* strains were isolated from various sources, with different antimicrobial resistance genes detected, including beta-lactamase gene *blaZ*, *iblaI*, methicillin resistance gene *mecA*, vancomycin resistance gene *vanB*, macrolide resistance gene *msr (A)*, tetracycline resistance genes *tet (K)* and *tet (M)*, chloramphenicol resistance gene cat, macrolide/clindamycin resistance gene *remA /C*, tobramycin resistance gene *aaaD*, and metallothiol transferase resistance gene *fosB*. These genes were detected using DNA microarray systems.^62^

*Salmonella* contamination was found in 81 RTE salads and vegetables, with resistance to sulfonamide antibiotics due to *sul1*, *sul2*, and *sul3* resistance genes, detected via polymerase chain reaction.^36^

*Listeria monocytogenes* was isolated from various RTE foods, and the antibiotic resistance genes *fosX, norB, mprF*, and *lin* were found, conferring resistance to antibiotics such as Fosfomycin, quinolones, and lincosamides. *tetA* and *tetC* genes provided resistance to tetracycline antibiotics, and the resistance genes had different mechanisms.^63^ In RTE meat products, including hamburger and raw kebab, *E. coli*, *S. aureus*, *L. monocytogenes*, and *Salmonella* spp. were detected, with resistance genes like *blaSHV, blaZ, blaTEM*, and *mecA* identified using PCR.^47^

 Dairy products made from raw or bovine milk contained pathogens like *S. aureus*, *L. monocytogenes*, *Salmonella* spp., and *E. coli*, with antibiotic resistance genes like *blaTEM, blaSHV, blaZ*, and *mecA* identified in foodborne bacteria isolated from bovine milk.^64^ The prevalence of these genes varied in different pathogens. Resistance gene subtypes were also mentioned, with different genes identified in RTE meat, vegetables, and fruit. For example, *catA1* was the most abundant ARG in RTE meat, followed by *bacA, tetM* and *tetA* were also present in RTE meat. In RTE vegetables, genes like *mexF, mexB, mexW, acrB, acrA, emrD*, and *tolC* were found, and in RTE fruit, genes like *acrB, acrA, emrD, tolC, mdtH, mdfA, bacA*, and *Bcr* were identified.^65^

## Effects of drug resistance food pathogen in RTE food

 Drug resistance occurs when various microorganisms like bacteria, fungi, viruses etc. adapt themselves in the presence of any drug or antibiotics and thus causes serious threats to public health. The effect of drug resistance in human health includes increase mortality rate among humans, increase morbidity rate, increased risk of transmission and dissemination, as well as higher healthcare costs, reduced efficacy of related antibiotics used in humans.^66^

 The resistant bacteria against drugs or antibiotics will increases the serious health issues and increase the chances of death among humans. According to a global status because of the drug resistant infections each year 700 000 people lose their lives.^48^ In an CDC (Center for Disease Control and Prevention) study mortality rate data among two countries i.e., United States and Europe were compared from which the number of affected people from drug resistance were 2 million approx. in both the countries and the number of mortality rate in the United States was 23 000 and in Europe. If no effective measure will be taken against drug resistance, it was estimated that about 10 million people will die by 2050 globally.^66^ According to a European study, because of drug resistance, more than 33 000 people die in Europe each year. Drug resistant infections also increases the morbidity rate among humans and prolonged stays in hospital which are infectious and spread their infections to other people.^67^ It also increases the healthcare costs among humans as the patients need isolated beds, prolonged hospitalization, ICUs, additional antibiotics, more diagnostic test to prevent the spread of infection. In the United States for treating the person with drug resistant infection, the hospital bill adds about $1400 and it goes upto more than $2 billion every year this was according to CDC data.^66^ This healthcare cost increases globally by 2050 from $300 billion to $1 trillion annually. Apart from serious health issue caused by drug resistant infection in humans, it also has global economic impact. Due to global economic impact, it was estimated that by 2050, 7% of GDP will be lost and because of this it is more difficult for developing countries to overcome this situation. And if actions have not taken there is an adverse impact on economy worldwide.^68^ According to CDC, in US the cost of drug resistance infection is $55 billion from which $20 billion for healthcare and $35 billion for lost productivity. A study showed that global GDP decrease to 1% and by 2050 the GDP decreased to 5-7%. Among pathogenic bacteria there is a antibiotic resistance is growing which was a major risk to the public’s health.^66^

## Methods to regulate drug resistance in RTE food

 In the context of RTE foods, their convenience makes them high-risk for foodborne outbreaks if mishandled or stored at improper temperatures, promoting bacterial growth. To ensure the safety of RTE foods, it is essential to regulate bacteria concentrations.^69^ Drug resistance in RTE foods can be mitigated through Good Manufacturing Practices (GMPs), which encompass proper handling, storage, cleaning, disinfection, and hygiene practices in food production facilities.^70^

 Additionally, preventing contamination of raw materials, especially those originating from animals or prone to cross-contamination by animal feces, requires various preventive controls.^71^ These include ensuring suppliers adhere to appropriate agricultural practices, utilizing kill-step methods like irradiation and pasteurization, and conducting environmental sampling to eliminate pathogens in the post-processing environment.^72^

 Furthermore, implementing Good Agricultural Practices (GAP) in animal and plant production, as well as adopting Hazard Analysis and Critical Control Points (HACCP), can play vital roles in ensuring food safety throughout the production, distribution, and consumption of RTE foods.^72^ Regulations limiting antibiotic use in animal agriculture, particularly in RTE foods, can also reduce the emergence of antibiotic-resistant bacteria.

 Consumers’ preference for antibiotic-free meat, along with other measures like improved animal housing, immunization, and hygiene, can help decrease antibiotic usage in animals. The direct or indirect transmission of antibiotic-resistant illnesses to humans through food consumption emphasizes the need to prohibit the use of antibiotics for growth promotion and enforce rules for their therapeutic administration in animals. Employing immunizations, herbal medicines, enzyme preparations, and other techniques can reduce antibiotic use while enhancing animal health.^73^

 To control drug resistance in RTE foods, it is crucial to identify potential sources of contamination, implement remedial actions, and conduct testing for antibiotic-resistant bacteria. Educational programs targeting both consumers and food workers can promote proper food safety procedures.^74^ Moreover, alternatives to antibiotics, such as natural antimicrobials and probiotics, can be employed to limit bacterial growth and reduce the risk of drug resistance in RTE foods.^51^

## Conclusion

 In conclusion, the uptake of RTE food has become a component of our daily routine, especially for those who have busy lifestyles. However, the convenience of such food comes at a cost. Studies have shown that the regular consumption of these foods is contributing to the increase in multidrug resistance in humans. The use of antibiotics in the production of these foods, along with poor hygiene practices during their preparation, has led to the appearance of resistant bacteria, which is a serious public health concern. It is imperative that we take measures to reduce the consumption of RTE food and promote healthy eating habits. This can be achieved by increasing awareness about the risks associated with the consumption of such food and encouraging people to adopt healthy food choices. Additionally, proper regulation of the use of antibiotics in food production, along with good hygiene practices during food preparation, can go a long way in reducing the spread of antibiotic-resistant bacteria. Overall, the issue of multidrug resistance in humans because of the uptake of RTE food is a complex problem that requires a multifaceted solution. The public, the food industry, and the government all have a role to play in addressing this issue and ensuring that we can continue to enjoy convenient food options while safeguarding our health.

## Future prospects

 The demand for frozen food is increasing as the RTE food market expands because it takes less time to prepare, requires little to no heat, can be consumed at any time, such as during breakfast, lunch, or dinner, and has a longer shelf life, making it simple to store for future use. This is because there are more people working in developed countries and because their lives are busier and more hectic. When kept at room temperature in a sealed container, frozen RTE food is shelf-stable and is prepared for consumption. Because it is more convenient for people to shop for groceries online these days, customers bought RTE food online because it is simple to choose and there is no need to go outside to get food. The RTE market will expand more in the future as a result of expanding urbanization and people’s growing knowledge of the advantages of frozen meals. Since artificial preservatives can hurt our bodies, consumers want RTE foods that are lightly processed and have a longer shelf life without losing quality food is in such high demand that the market’s growth curve will incline upward. More quality RTE food items, such meals inspired by different cuisines, are already available on the market as the population’s preference for RTE food grows.

## Competing Interests

 None.

## Ethical Approval

 Not applicable.
